# Supplementary material for: MEIOC prevents continued mitotic cycling and promotes meiotic entry during mouse oogenesis
Source: Development. 2026 Jan 12;153(1):dev205037. doi: 10.1242/dev.205037 (PMC12848572; doi:10.1242/dev.205037)
Supplement: Supplementary information [file develop-153-205037-s1.pdf]

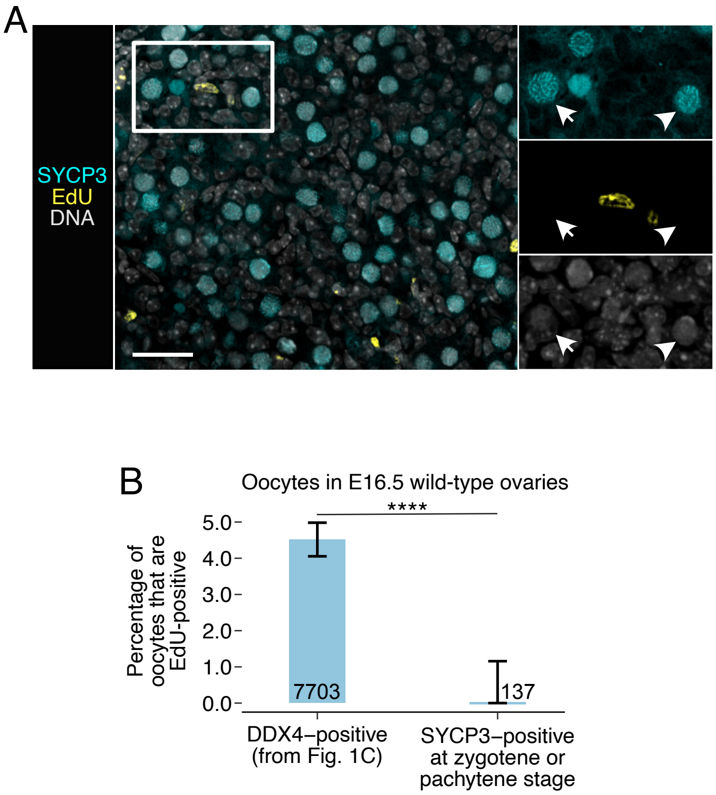

Fig. S1. EdU does not localize to oocytes undergoing double strand break repair during meiotic prophase I.

(A) Absence of EdU signal in SYCP3-positive oocytes in zygotene (arrowhead) and pachytene (arrow) stages of meiotic prophase I in wild-type ovaries at E16.5. Scale bar = 25.0µm in large panel and 17.4µm in insets.

(B) Percentage of EdU-positive oocytes in wild-type ovaries at E16.5. Data from DDX4, which labels all oocytes, is from Figure 1C. SYCP3 was used to identify oocytes at zygotene or pachytene stages of meiotic prophase I. Error bars represent 95% confidence interval. Number on or adjacent to bar represent total number of oocytes quantified. DDX4 dataset was collected from 5 embryos. SYCP3 dataset was collected from 3 embryos.

****, adj. $P<0.0001$; See Table S1 for statistical details.

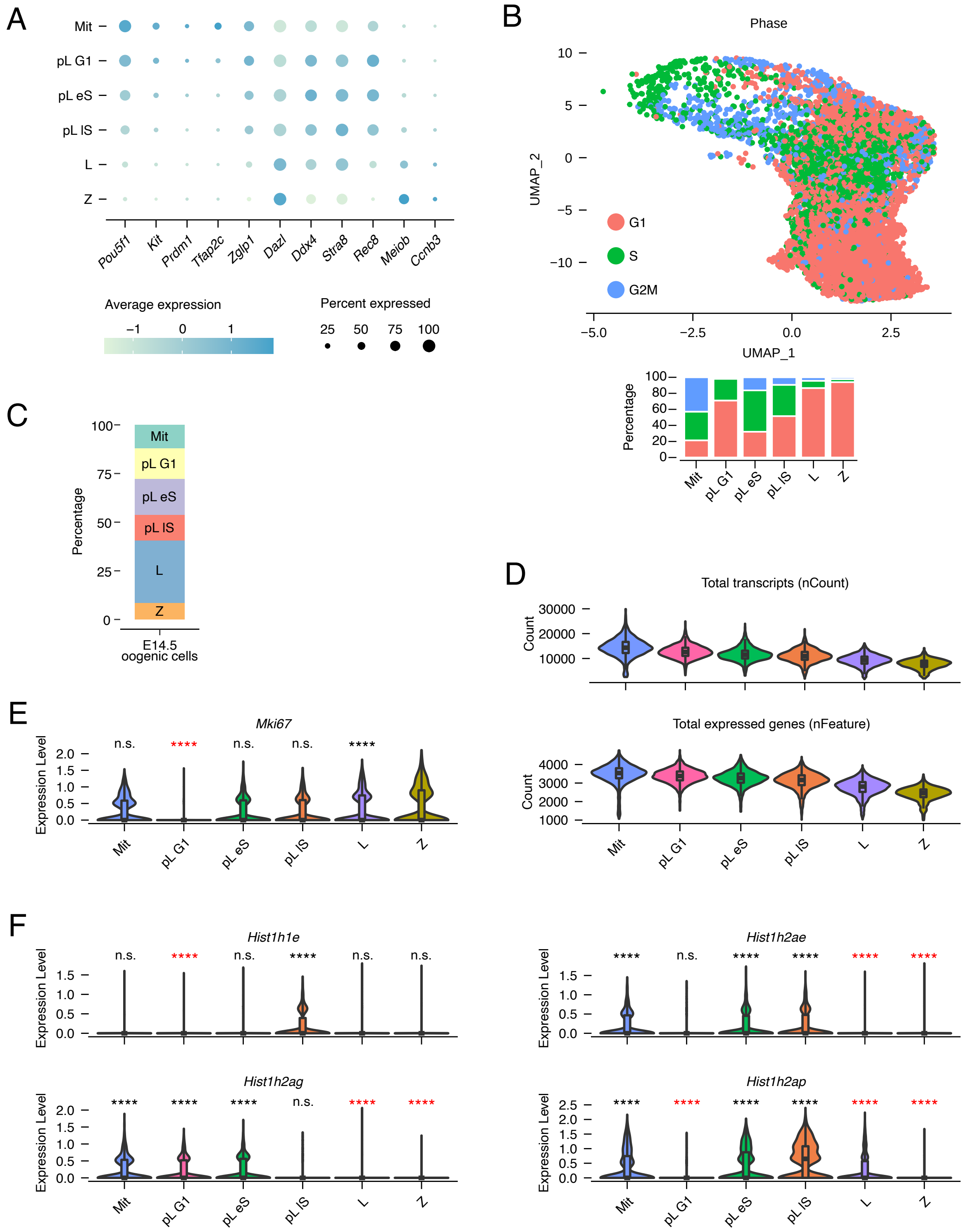
A
Mit
pL G1
pL eS
pL lS
L
Z
Pou5f1
Kit
Prdm1
Tfap2c
Zglp1
Dazl
Ddx4
Stra8
Rec8
Meiob
Ccnb3
Average expression
Percent expressed
B
Phase
G1
S
G2M
UMAP_1
UMAP_2
Percentage
C
Percentage
E14.5 oogenic cells
D
Total transcripts (nCount)
Total expressed genes (nFeature)
Count
E
Mki67
Expression Level
n.s.
****
F
Hist1h1e
Hist1h2ae
Hist1h2ag
Hist1h2ap

Fig. S2. Identification of E14.5 oogenic cell subpopulations across the mitosis-to-meiosis transition via scRNA-seq.

(A) Dotplot of expression levels and percentage of cells for markers used to assign clusters. Data from scRNA-seq of *Pou5f1*:EGFP-positive cells from E14.5 ovary (Zhao et al., 2020).

(B) UMAP and per-cluster barplot of Seurat-based cell cycle phase designations used to assign clusters.

(C) Percentage of each oogenic cell type identified, relative to all oogenic cells.

(D) Total number of transcripts detected (nCount) and genes expressed (nFeature) in oogenic cell clusters.

(E) pL G1 cluster exhibits depleted *Mki67*, which is at its lowest during G1 phase of the cell cycle and thereby supports the cluster's G1 phase designation.

(F) pL eS and lS clusters exhibit enrichment of replication-dependent histones *Hist1h1e*, *Hist1h2ae*, *Hist1h2ag*, and *Hist1h2ap*, which confirms the clusters' S phase designation.

****, adj. P<0.0001; n.s., not significant. Red and black asterisks mark statistical depletion and enrichment, respectively.

Table S1. Supplemental data for Fig. 1 and Fig. S1.

Available for download at
https://journals.biologists.com/dev/article-lookup/doi/10.1242/dev.205037#supplementary-data

Table S2. Supplemental data for Fig. 2.

Available for download at
https://journals.biologists.com/dev/article-lookup/doi/10.1242/dev.205037#supplementary-data

Table S3. Supplemental data for Fig. S2.

Available for download at
https://journals.biologists.com/dev/article-lookup/doi/10.1242/dev.205037#supplementary-data

Table S4. Supplemental data for Fig. 3.

Available for download at
https://journals.biologists.com/dev/article-lookup/doi/10.1242/dev.205037#supplementary-data

Table S5. Supplemental data for Fig. 4.

Available for download at
https://journals.biologists.com/dev/article-lookup/doi/10.1242/dev.205037#supplementary-data

Table S6. Supplemental data for Fig. 5.

Available for download at
https://journals.biologists.com/dev/article-lookup/doi/10.1242/dev.205037#supplementary-data
